# Leaf senescence characteristics and economic benefits of rice under alternate wetting and drying irrigation and blended use of polymer-coated and common urea

**DOI:** 10.3389/fpls.2024.1444819

**Published:** 2024-12-24

**Authors:** Dongliang Qi, Si Chen, Wenjun Yue, Yonggang Duan

**Affiliations:** ^1^ School of Hydraulic Engineering, Zhejiang University of Water Resources and Electric Power, Hangzhou, China; ^2^ Engineering Research Center of Ecology and Agriculture Use of Wetland, Ministry of Education, Yangtze University, Jingzhou, Hubei, China

**Keywords:** economic benefits, high-efficiency N fertilizer, leaf senescence, rice, water-saving irrigation

## Abstract

Water-saving irrigation and the mixed application of controlled-release nitrogen fertilizer (CRNF) and common urea (CU; with a higher nitrogen release rate) have shown promise in improving rice yield with high resource use efficiency. However, the physiological mechanism underlying this effect remains largely unknown. This study involved a field experiment on rice in Jingzhou City, Central China, in 2020 and 2021. Two irrigation regimes were employed [alternate wetting and drying irrigation (AWD) and conventional flood irrigation (CI)], with three nitrogen (N) compounding modes [00% CU (N1), 60% CRNF + 40% CU (N2), and 100% CRNF (N3)] with an equal N rate of 240 kg ha^−1^. The results indicated a significant interactive effect of watering regimes and N compounding modes on net photosynthetic rate (*P*_n_), leaf area index (LAI), and *SPAD* values; activities of superoxide dismutase (SOD), peroxidases (POD), catalase (CAT), glutamine synthetase (GS), glutamine 2-oxoglutarate amidotransferase (GOGAT), and nitrate reductase (NR); and the contents of malondialdehyde (MDA) and soluble protein in rice leaves. Compared with N1, N2 and N3 increased the *P*_n_, LAI, and *SPAD* values; activities of SOD, POD, CAT, NR, GS, and GOGAT; and soluble protein content but decreased MDA content in the post-growth (heading and maturity) stages by 8.7%–31.2% under the two irrigation regimes. Compared to CI (traditional irrigation), AWD had higher *P*_n_, LAI, and *SPAD* values; activities of SOD, POD, CAT, NR, GS, and GOGAT; and soluble protein content (increased by 12.1%–38.0%, and lower MDA content (reduced by 13.1%–27.6%) irrespective of N compounding modes. This suggested that AWD combined with N2 and N3 could delay the leaf senescence of rice, thus achieving a larger grain yield. Moreover, AWD significantly decreased water costs (irrigation amount) and labor costs (irrigation frequency), thus increasing total income. N2 decreased fertilizer costs with a higher or comparable total income compared with N3. Therefore, the AWDN2 treatment achieved the highest net income (13,907.1 CNY ha^−1^ in 2020 and 14,085.7 CNY ha^−1^ in 2021). AWD interacted with 60% CRNF + 40% (N2) to delay leaf senescence by improving photosynthesis, antioxidant defense system, osmoregulation, and N assimilation, contributing to high grain yield and net income in rice.

## 1 Introduction

Rice (*Oryza sativa* L.) is a primary cereal crop serving as the staple diet for approximately 50% of the world’s population. With the increasing world population, the rice yield needs to be boosted by 25% to satisfy growing food demand (Fertitta-Roberts et al., 2019; Su et al., 2023). The production of rice is influenced by soil characteristics (Bueno et al., 2010), cultivation measurement (Ly et al., 2016), and weather conditions (Chiba et al., 2017). Notably, freshwater plays a vital role in determining the physiological processes of rice. Rice cultivation consumes a substantial amount of freshwater resources, with over two-thirds of the irrigation water in China being used for rice cultivation (National Bureau of Statistics, 2022). A previous study has estimated that 30–40 million ha of rice crops will face water scarcity by 2050 (Mboyerwa et al., 2021). Moreover, climatic changes, urbanization, and industrialization have led to a rise in the use of valuable freshwater resources, intensifying the water scarcity for rice cultivation (Ishfaq et al., 2020). Therefore, water-saving irrigation strategies with high water use efficiency (WUE) need to be explored to ensure sustainable rice production.

In addition to irrigation water, the application of nitrogen (N) fertilizer is another important factor affecting rice production, constituting a major resource input for rice crops (Spiertz, 2010; Wang et al., 2018). The application of N fertilizer enhances rice yield (>30%) and is involved in 40% of the rice growth processes (Peng et al., 2021). Applying a high amount of N fertilizer increases grain yield, but low N use efficiency (NUE) is a major concern for intensive rice production (Ding et al., 2018). Low NUE is also accompanied by various environmental issues, including greenhouse gas emissions, water eutrophication, and soil degradation (Ju et al., 2009; Spiertz, 2010; Ding et al., 2018; Qi et al., 2023a). In addition to adverse environmental impacts, the limited yield gain at higher N application rates is a concern, decreasing the economic profits of farmers (Yuan et al., 2022). Moreover, the excessive or non-scientific application of N fertilizer reduces the grain yield of rice (Wang et al., 2016; Xu et al., 2018). N and water work together to determine the physiological processes and final yield of rice. Therefore, efficient management of irrigation water and N fertilizer should be encouraged to synergistically enhance grain yield and water–N use efficiencies of rice cultivation.

Various water-saving irrigation technologies, such as internal drainage (Ramasamy et al., 1997), continuous soil saturation (Borrell et al., 1997), rainfall-adapted irrigation (Yan et al., 2022), intermittent irrigation (Berkhout et al., 2015), non-flooded mulching cultivation (Zhou et al., 2023), and alternate wetting and drying irrigation (AWD) (Belder et al., 2004; Ishfaq et al., 2020; Liang et al., 2023), have been explored to address water shortages in rice cultivation. Of these, AWD is the most popular irrigation strategy because it is simple to operate, easy to follow, and less labor intensive. Moreover, this kind of strategy provides a comparable or even larger grain yield of rice with obviously less volume of irrigation water (Lampayan et al., 2015; Xu et al., 2018; Ishfaq et al., 2020; Zhang et al., 2021; Su et al., 2023). Improved N fertilizer management strategies have also been investigated to improve NUE in rice production. Techniques such as deep placement of N fertilizer, site-based N fertilization, multi-split N management, and precise and smart N application have demonstrated the potential to enhance NUE and grain yield in rice (Liu et al., 2013; Hooper et al., 2014; Ding et al., 2018). In particular, deep placement of urea significantly increased rice yield and NUE and reduced cumulative methane and nitrous oxide emissions compared with broadcast urea (Gaihre et al., 2015; Islam et al., 2022). However, the application of these N management strategies typically relies on knowledge-intensive technologies, specialized equipment, and substantial labor forces, limiting their application (Wang et al., 2018). Alternately, highly efficient N fertilizers, such as controlled-release N fertilizer (CRNF), have shown promise in better meeting N demand and enhancing the growth processes of rice (Song et al., 2014; Zheng et al., 2016; Ding et al., 2018; Li et al., 2022). Additionally, CRNF fertilization is easy to implement and requires low labor costs, making it a hot topic of research both domestically and internationally. The application of CRNF can synergistically improve WUE and NUE compared with common urea (CU) fertilization (Ye et al., 2013; Ding et al., 2018; Sun et al., 2019; Yan et al., 2022; Zhang et al., 2024). However, pure (100%) application of CRNF cannot meet the N demands of rice in the early (tillering) stage (Zhang et al., 2021). Additionally, the material used for CRNF production is expensive, reducing the possibility of adoption by farmers for grain crop production, especially in developing countries (Shaviv, 2001). Therefore, blending the application of CRNF and CU is a newly developed N management strategy for the sustainable development of rice production (Zhang et al., 2021; Yan et al., 2022).

Plants regulate their cellular metabolism and defense mechanisms in the face of drought, waterlogging, salinity, and other abiotic stresses (Bohnert and Jensen, 1996). Superoxide dismutase (SOD), peroxidases (POD), and catalase (CAT) are extremely important protective enzymes involved in the active oxygen metabolism in plants; they scavenge oxygen free radicals in biological systems (Foyer and Noctor, 2000). Malondialdehyde (MDA) is a stable product of membrane lipid peroxidation, and its content can be used to assess the degree of oxidative damage (Ren et al., 2023). Soluble proteins are the main constituent of various cells and organelles, playing a vital role in photosynthesis (Qi et al., 2021). Chlorophyll is the most important and effective pigment required for normal photosynthesis in plants; its content (indicated by the leaf *SPAD* value) is closely associated with the degree of leaf senescence (Yu et al., 2023). The activities of SOD, POD, and CAT, along with the contents of MDA, soluble proteins, and *SPAD* values, are key physiological and biochemical indicators reflecting leaf senescence (Foyer and Noctor, 2000; Yu et al., 2023). An imbalance in active oxygen metabolism is one of the main causes of leaf aging. Drought, N deficiency, and their combination can reduce the activities of SOD, POD, and CAT, leading to the accumulation of reactive oxygen species (ROS), an increase in MDA content, chlorophyll degradation, reduced phytase activity, and diminished photosynthetic capacity, thus accelerating leaf senescence (Li et al., 2020; Qi et al., 2021). Implementing scientific irrigation regimes and N fertilization strategies are two effective approaches used to improve rice yield and resource use efficiency by influencing physiological processes (Sun et al., 2012; Wang et al., 2018). Compared with conventional flood irrigation (CI), moderate AWD increases the activities of GS, GOGAT, NR, SOD, POD, and CAT in rice leaves and increases net photosynthetic rate (*P*_n_) and *SPAD* value, thereby delaying leaf senescence (Fu et al., 2024). Li et al. (2010) also demonstrated that compared with full irrigation, deficit irrigation (alternate partial root-zone irrigation) increased chlorophyll content and SOD and POD activities but reduced MDA content in maize leaves. Moreover, compared with CU fertilization, the application of CRNF increased LAI, *P*_n_, and *SPAD* values (Wang et al., 2018) and the activities of GS, GOGAT, and NR in rice leaves (Yang et al., 2012). In terms of the water–N coupling effects, AWD attenuated the positive effects of moderate N dosages on the physiological and morphological characteristics of roots (Xu et al., 2018). AWD, when combined with an improved N fertilization mode (reducing N application rates by 20% and delaying N topdressing), enhanced the growth of both roots and shoots in rice plants (Yan et al., 2022). Moderate deficit irrigation interacted with CRNF to maintain the leaf greenness of maize by enhancing the LAI, *SPAD* value, and *P*_n_ (Li et al., 2020). However, the data on the effects of AWD combined with the blended use of CRNF and CU on leaf senescence characteristics and economic benefits in rice are still lacking.

The conventional N application mode usually leads to low NUE under water-saving irrigation conditions (Han et al., 2014). Therefore, clarifying whether a combination of CRNF (compounding CRNF and CU) and AWD can realize the goal of improving rice yield and resource use efficiency is essential (Qi et al., 2023a, b). Also, the physiological mechanism underlying the effect of this combination needs to be illustrated. Therefore, the present study evaluated the LAI, NR, GS, GOGAT, SOD, POD, and CAT activities; *P*_n_ and *SPAD* values; soluble protein and MDA contents; and economic benefits in rice production regarding the combination of CRNF and CU patterns under AWD compared with the CI and clarified the causes of possible differences. It was hypothesized that AWD interacted with the blended use of CRNF and CU to provide proper soil N and water conditions, thus delaying the senescence by improving photosynthesis, antioxidant defense system, osmoregulation, and N assimilation, and thus leading to a greater total income (grain yield) with less costs on resource input (water, N fertilizer, and labor), consequently achieving a high net income.

## 2 Materials and methods

### 2.1 Experimental site and materials

**Figure 1:**
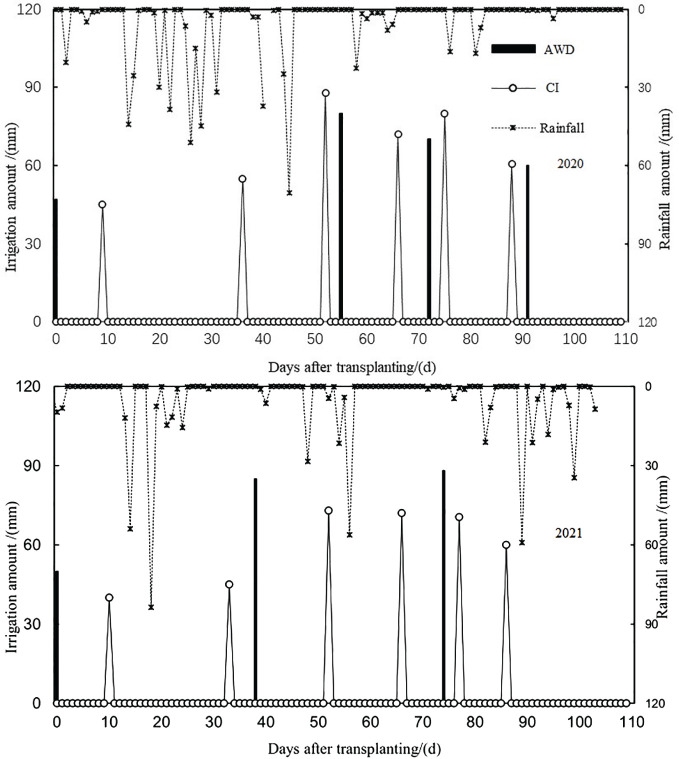
The volume of precipitation and irrigation water during the two rice-growing seasons (2020 and 2021) under conventional flooding irrigation (CI) and alternate wetting and drying irrigation (AWD).

Field experiments were conducted in Jingzhou City, Central China (latitude 30° 26′N, longitude 112° 39′E, altitude 30 m) between 2020 and 2021. This region has a subtropical monsoon climate with an average annual precipitation of 1,050 mm. The soil in the tested field was classified as Entisol (Typic Fluvaquent, Entisol, US classification). The initial topsoil (0–40 cm) had a pH of 7.3. The fertilizer characteristics of the soil were as follows: available N, 79.8 mg kg^−1^; total N, 2.10 g kg^−1^; Olsen P, 38.8 mg kg^−1^; total P, 0.48 g kg^−1^; total K, 0.87 g kg^−1^; and exchangeable K, 108.8 mg kg^−1^. The field moisture capacity and soil bulk density were 0.35 cm^3^ cm^−3^ and 1.41 g cm^−3^, respectively. The rainfall during the rice-growing season in the 2 years is shown in **Figure 1**.

### 2.2 Experimental design

The variety Liangyou 152 (*O. sativa* L.), which is now widely cultivated in the local area, was adopted as the tested crop. A 2 × 3 factorial design (two irrigation regimes and three N compounding modes) was conducted in a randomized complete block setup with three replications. The irrigation regimes consisted of CI and AWD. They were started 10 days after transplanting (DAT) and continued until the maturity of rice. A continuous layer (10–60 mm) of water was maintained in the CI plots after transplanting until the final drainage was approximately 10 days before the harvest. In the AWD plots, rice plants were flooded intermittently, and the irrigation (20–40 mm of water) was not conducted until the soil water potential (SWP) reached −10 kPa at 15−20-cm soil depth (Wang et al., 2016). The SWP was monitored using a tensiometer (5-cm sensor). Moreover, the irrigation was initiated for N fertilization and pesticide application. A flow meter (LXSG-50 Flow Meter; Shanghai Water Meter) was used for measuring the amount of irrigation water. The irrigation and drainage were conducted independently.

The N compounding modes consisted of 100% CU (N1), 60% CRNF + 40% CU (N2), and 100% CRNF (N3), with an equal N rate of 240 kg ha^−1^. Polymer-coated urea containing 43% N (Kingenta Ecological Engineering Co., Ltd., Shandong, China) was used as CRNF (N discharge continued for 90 days). The conduction of N1 exactly followed the N management strategy of local farmers: CU (prilled urea containing 46.4% N) was applied as a basal dose (60%) and as a topdressing in the tillering stage (40%). In N2 and N3, both CRNF and CU were applied once before transplantation. In addition, 529 kg ha^−1^ calcium superphosphate (P_2_O_5_ 17%) and 300 kg ha^−1^ muriate of potash were applied before sowing. Tillering N application was conducted on May 24 and 25. Four-week-old seedlings were transplanted on May 15 and 16 at a density of 24 hills m^−2^ (3 plants hill^−1)^. The rice plants were harvested on September 16–19 in the 2 years. The individual plots (8 × 5 m^2^) were edged with a plastic film inserted 30 cm below the soil surface to form a barrier. Diseases, insects, and weeds were well controlled across the two growing seasons.

### 2.3 Measurements

#### 2.3.1 LAI and *SPAD* values

The leaf area and *SPAD* values were collected at the tillering, jointing, heading, filling, and maturity stages, corresponding to 17 and 18, 41 and 42, 75 and 75, 96 and 97, and 125 and 126 DAT in 2020 and 2021, respectively. Leaf area was measured using an area meter (LI-3050C; Li-Cor, Lincoln, NE, USA) on green leaves from eight hills. The LAI was calculated as the ratio of total leaf area to per unit ground area (Li et al., 2020). *SPAD* values were determined using a portable SPAD-502 chlorophyll meter (Minolta Camera Co., Osaka, Japan).

#### 2.3.2 Physiological measurements

The functional leaves (last fully developed leaves) of rice were measured from three randomly selected plants in the jointing, heading, and maturity stages on the same date as leaf area determination. The *P*_n_ was measured between 10:00 AM and 1:00 PM on clear days using a portable photosynthesis system (LI-6400; Li-Cor Inc., NE, USA) when the photosynthetically active radiation above the canopy was 1,500 μmol m^−2^ s^−1^. The POD, SOD, and CAT activities were determined using guaiacol colorimetry, nitro blue tetrazolium, and permanganate titration methods, respectively, as previously described (Ren et al., 2018). The MDA content was measured using the thiobarbituric acid method (Du and Bramlage, 1992). The NR, GS, and GOGAT activities of functional leaves were determined using the protocols proposed by Aslam et al. (2001); Lea et al. (1990), and Singh and Srivastava (1986), respectively. The soluble protein content was determined using the method proposed by Mohammadkhani and Heidari (2007).

#### 2.3.3 Grain yield

An 8-m^2^ area of rice plants in the middle of each plot was harvested for grain yield determination (the grains were air-dried).

### 2.4 Analysis of economic benefit

The economic benefit of rice was computed using the following formula proposed by Lai et al. (2022):


(1) TI=RP×GY

(2) NI=TI–FC–SC–WC−OC

where TI is the total income (CNY ha^−1)^, RP is the rice price (CNY kg^−1^), GY is the grain yield (kg ha^−1^), NI is the net income (CNY ha^−1^), FC is the fertilizer cost (CNY ha^−1^), LC is the labor cost (CNY ha^−1^), WC is the water cost (CNY ha^−1^), and OC is the other cost (CNY ha^−1^, including costs of seed, weeding, pest control, and field management).

### 2.5 Statistical analysis

The analysis of variance was performed using the SAS/STAT statistical analysis package (version 6.12; SAS Institute, Cary, NC, USA). The statistical model used in the study accounted for sources of variation due to replication, year, irrigation regime, N compounding mode, and their interactions (year × irrigation regime, year × N compounding mode, and year × irrigation regime × N compounding mode). The data for each sampling date were analyzed separately, and means were compared using the least significant difference (LSD) test at *p* < 0.05 (LSD_0.05_).

## 3 Results

**Figure 2:**
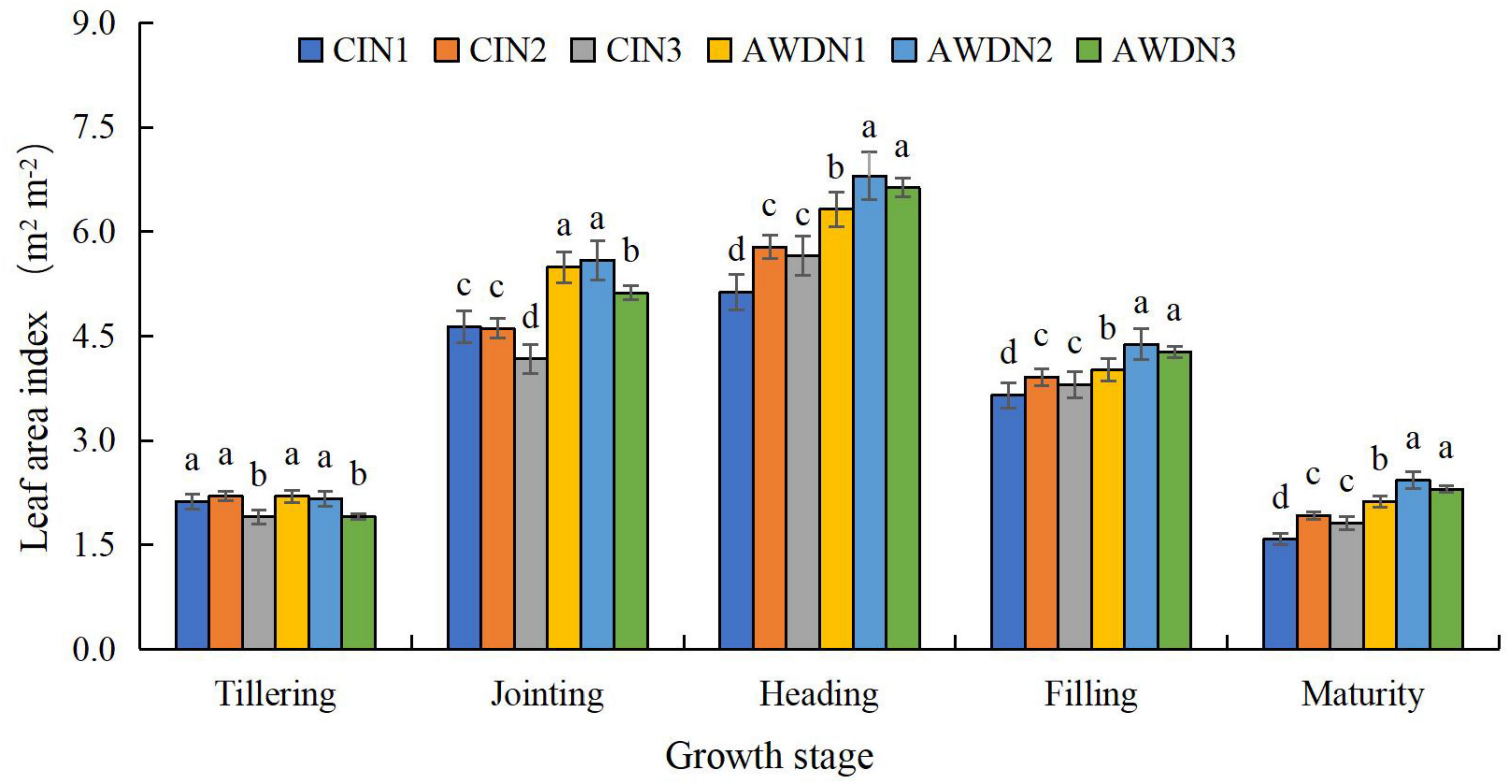
Leaf area index (LAI) of rice as affected by different water and nitrogen management strategies. CI and AWD represent conventional flooding irrigation and alternate wetting and moderate drying irrigation, respectively. N1, N2, and N3 represent 100% CU, 60% CRNF + 40% CU, and 100% CRNF at an equivalent N rate of 240 kg ha^−1^, respectively. Values (mean ± standard error, n = 6) are mean of 2 years and three replicates. Means by different letters are significantly *p* < 0.05.

**Figure 3:**
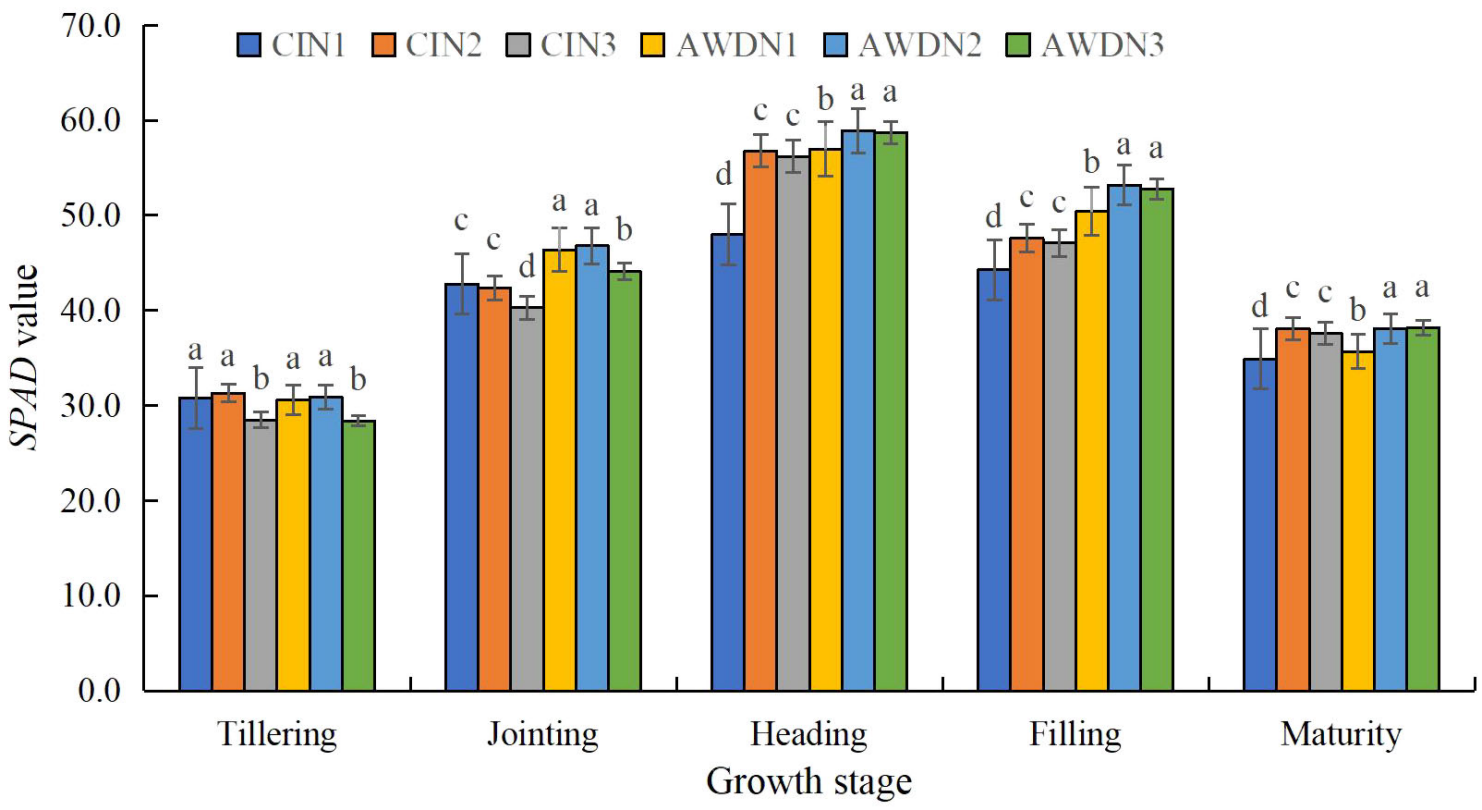
*SPAD* values of rice leaves as affected by different water and nitrogen management strategies. CI and AWD represent conventional flooding irrigation and alternate wetting and moderate drying irrigation, respectively. N1, N2, and N3 represent 100% CU, 60% CRNF + 40% CU, and 100% CRNF at an equivalent N rate of 240 kg ha^−1^, respectively. Values (mean ± standard error, n = 6) are mean of 2 years and three replicates. Means by different letters are significantly different at *p* < 0.05.

**Figure 4:**
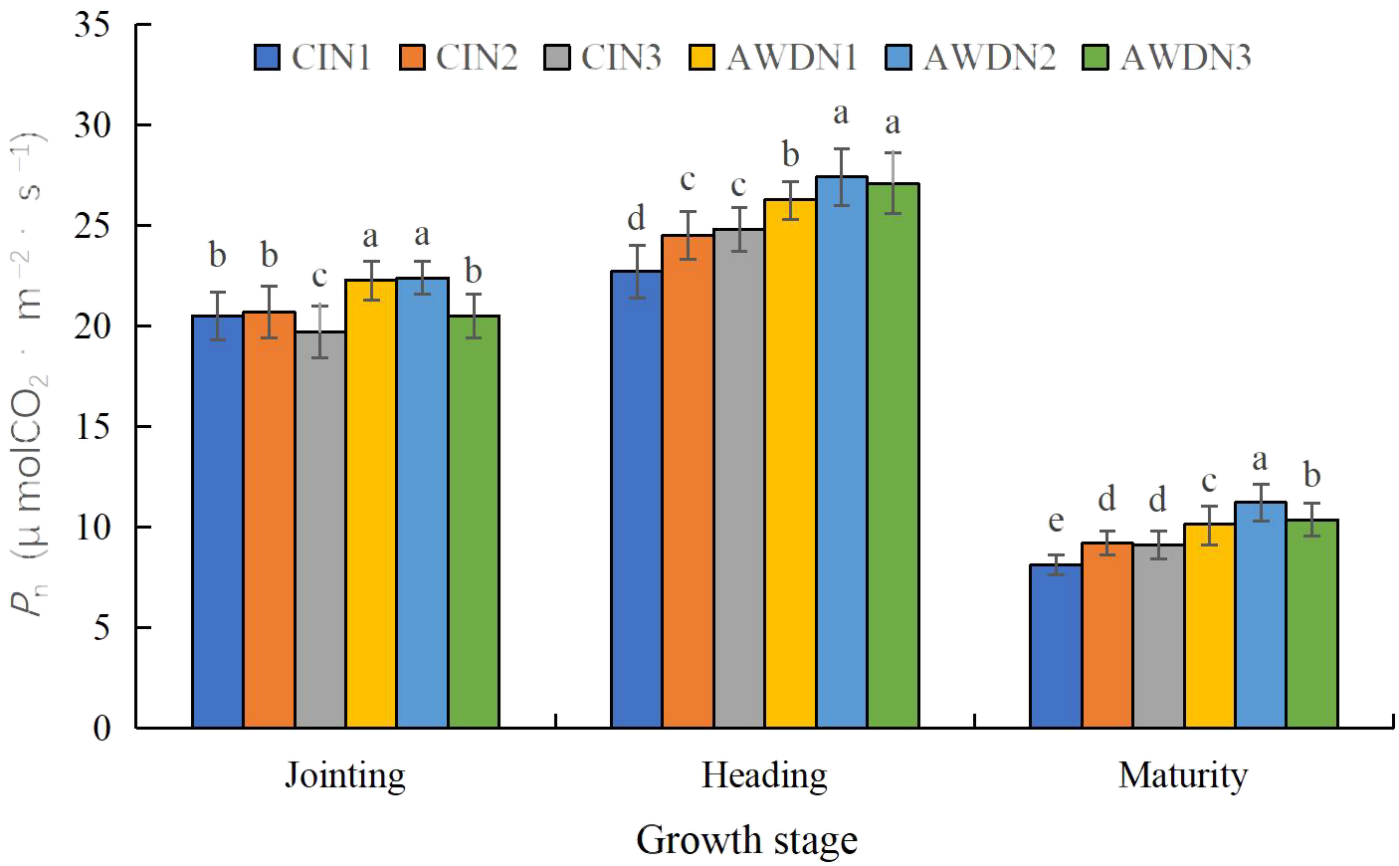
Net photosynthetic rate (*P*_n_) of rice leaves as affected by different water and nitrogen management strategies. CI and AWD represent conventional flooding irrigation and alternate wetting and moderate drying irrigation, respectively. N1, N2, and N3 represent 100% CU, 60% CRNF + 40% CU, and 100% CRNF at an equivalent N rate of 240 kg ha^−1^, respectively. Values (mean ± standard error, n = 6) are mean of 2 years and three replicates. Means by different letters are significantly different at *p* < 0.05.

**Figure 5:**
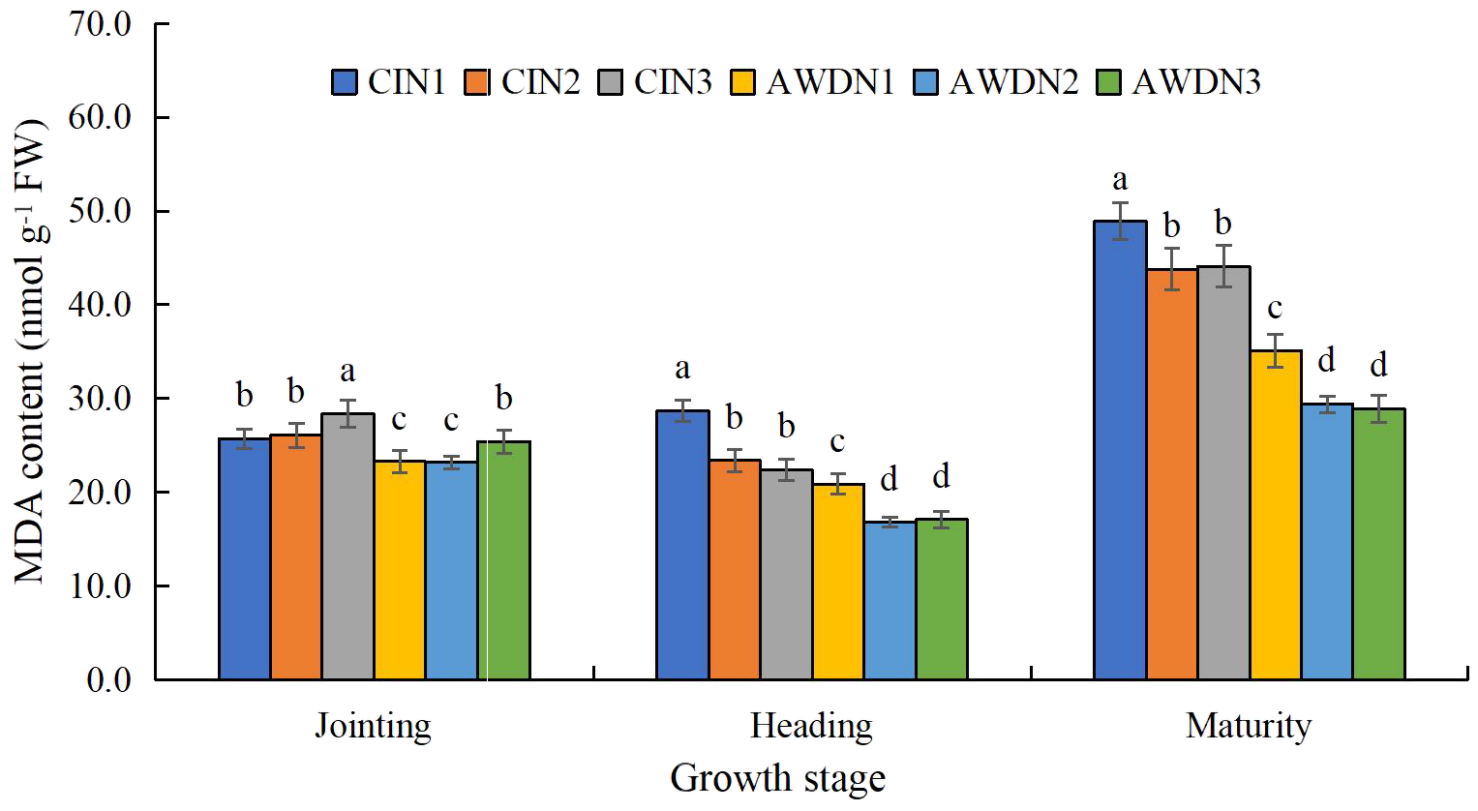
Malondialdehyde (MDA) content of rice leaves affected by different water and nitrogen management strategies. CI and AWD represent conventional flooding irrigation and alternate wetting and moderate drying irrigation, respectively. N1, N2, and N3 represent 100% CU, 60% CRNF + 40% CU, and 100% CRNF at an equivalent N rate of 240 kg ha^−1^, respectively. Values (mean ± standard error, n = 6) are mean of 2 years and three replicates. Means by different letters are significantly different at *p* < 0.05.

**Figure 6:**
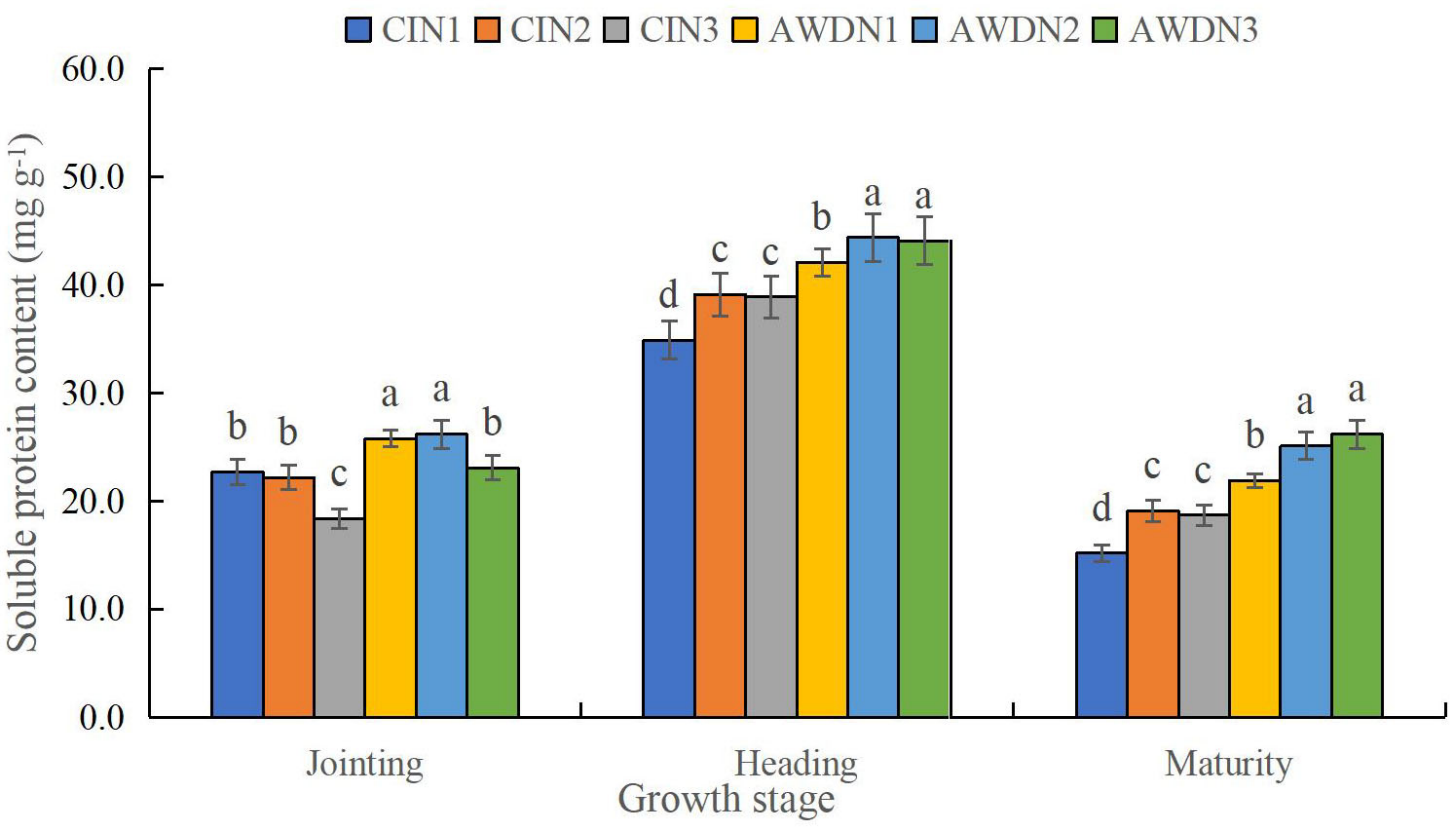
Soluble protein content of rice leaves affected by different water and nitrogen management strategies. CI and AWD represent conventional flooding irrigation and alternate wetting and moderate drying irrigation, respectively. N1, N2, and N3 represent 100% CU, 60% CRNF + 40% CU, and 100% CRNF at an equivalent N rate of 240 kg ha^−1^, respectively. Values (mean ± standard error, n = 6) are mean of 2 years and three replicates. Means by different letters are significantly different at *p* < 0.05.

Watering regime (W), N fertilization strategies, and W × N had significant or extremely significant effects on *P*_n_, LAI, and *SPAD* value; activities of SOD, POD, CAT, NR, GS, and GOGAT; and contents of MDA and soluble protein in rice leaves. However, no significant difference was observed in these parameters between the two study years (**Table 1**). Therefore, the aforementioned parameters were presented as mean across the two study years (**Tables 2**, **3**; **Figures 2**–**6**).

**Table 1:** Analysis of variance of *SPAD* value, superoxide dismutase (SOD), peroxidases (POD), catalase (CAT), nitrate reductase (NR), glutamine synthetase (GS), glutamine 2-oxoglutarate amidotransferase (GOGAT), net photosynthetic rate (*P*_n_), leaf area index (LAI), *SPAD* value, and malondialdehyde (MDA) and soluble protein contents under condition of irrigation regimes and nitrogen management strategies interaction.

Source of variation	Degree of freedom	*SPAD*	SOD (unit. g^−1^ FW min^−1^)	POD (ΔOD470 g^−1^ FW min^−1^)	CAT (μmol H_2_O_2_ g^−1^ FW min^−1^)	GS (μmol g^−1^ h^−1^)	NR (μg g^−1^ h^−1^)	GOGAT (μmol g^−1^ h^−1^)	LAI (m^2^ m^−2^)	*P*_n_ (μmol CO_2_ m^−2^ s^−1^)	MDA (μmol CO_2_ m^−2^ s^−1^)	Soluble protein content (mg g^−1^)
Y	1	NS	NS	NS	NS	NS	NS	NS	NS	NS	NS	NS
W	1	**	**	**	**	**	**	**	**	**	**	**
N	2	**	**	**	**	**	**	**	**	**	**	**
Y × W	1	NS	NS	NS	NS	NS	NS	NS	NS	NS	NS	NS
Y × N	2	NS	NS	NS	NS	NS	NS	NS	NS	NS	NS	NS
W × N	2	**	**	**	**	*	*	**	*	**	**	*
Y × W × N	2	NS	NS	NS	NS	NS	NS	NS	NS	NS	NS	NS

NS indicates statistical significance at *p* > 0.05 within a column. * and ** represent statistical significance at *p* < 0.05 and *p* < 0.01 respectively. Y, W, and N represent year, water, and nitrogen, respectively.

**Table 2:** Superoxide dismutase (SOD), peroxidases (POD), and catalase (CAT) of rice leaves at the jointing, heading, and maturity stages as affected by different water and nitrogen management strategies.

Treatment	SOD (unit. g^−1^ FW min^−1^)			POD (ΔOD470 g^−1^ FW min^−1^)			CAT (μmol H_2_O_2_ g^−1^ FW min^−1^)		
	Jointing	Heading	Maturity	Jointing	Heading	Maturity	Jointing	Heading	Maturity
CIN1	449.5 ± 14.3c	501.7 ± 14.3d	479.7 ± 12.3d	51.4 ± 1.9b	65.8 ± 1.4d	45.8 ± 1.0d	18.6 ± 0.8c	21.6 ± 0.8d	16.2 ± 0.5d
CIN2	421.8 ± 10.6c	571.8 ± 15.0c	541.8 ± 13.7c	53.6 ± 2.1b	73.4 ± 1.9c	51.4 ± 1.7c	18.4 ± 1.1c	28.4 ± 1.0c	19.4 ± 0.8c
CIN3	400.8 ± 15.4d	570.8 ± 17.8c	530.8 ± 12.5c	44.1 ± 20c	74.1 ± 2.5c	52.1 ± 2.1c	16.5 ± 0.8d	26.5 ± 0.9c	19.5 ± 0.6c
AWDN1	502.3 ± 21.1a	662.3 ± 25.1b	612.3 ± 25.1b	59.3 ± 2.6a	83.3 ± 4.6b	60.3 ± 2.6b	23.2 ± 1.0a	38.2 ± 1.3b	23.2 ± 0.7b
AWDN2	511.0 ± 18.9a	781.0 ± 28.9a	675.0 ± 21.9a	60.2 ± 1.8a	90.2 ± 5.8a	68.2 ± 4.7a	24.5 ± 1.1a	44.5 ± 2.1a	27.5 ± 1.1a
AWDN3	472.7 ± 19.5b	772.7 ± 31.5a	672.7 ± 22.1a	52.1 ± 1.7b	91.1 ± 3.6a	69.1 ± 3.9a	20.3 ± 0.9b	45.3 ± 1.9a	28.3 ± 1.2a

CI and AWD represent conventional flooding irrigation and alternate wetting and moderate drying irrigation, respectively. N1, N2, and N3 represent 100% CU, 60% CRNF + 40% CU, and 100% CRNF at an equivalent N rate of 240 kg ha^−1^, respectively. Values are mean of 2 years and three replicates, and values (means ± standard error, n =6) followed by different letters within a column are significantly different at the probability level of 0.05.

**Table 3:** Nitrate reductase (NR), glutamine synthetase (GS), and glutamine 2-oxoglutarate amidotransferase (GOGAT) of rice leaves at the jointing, heading, and maturity stages as affected by different water and nitrogen management strategies.

Treatment	NR (μg g^−1^ h^−1^)			GS (μmol g^−1^ h^−1^)			GOGAT (μmol g^−1^ h^−1^)		
	Jointing	Heading	Maturity	Jointing	Heading	Maturity	Jointing	Heading	Maturity
CIN1	97.5 ± 4.3c	102.7 ± 2.3d	51.7 ± 1.3d	151.4 ± 1.9b	165.8 ± 4.4d	80.8 ± 1.8d	25.6 ± 0.8c	21.6 ± 0.8d	16.8 ± 0.5d
CIN2	96.7 ± 3.6c	112.5 ± 4.0c	64.1 ± 2.7c	153.6 ± 2.1b	173.4 ± 5.9c	91.4 ± 2.7c	26.4 ± 1.1c	29.4 ± 1.0c	22.4 ± 0.8c
CIN3	87.8 ± 4.4d	110.8 ± 3.8c	62.8 ± 1.5c	144.1 ± 2.0c	174.1 ± 6.5c	92.1 ± 3.1c	21.5 ± 0.8d	31.5 ± 1.5c	21.5 ± 1.1c
AWDN1	122.3 ± 7.1a	124.3 ± 4.4b	74.3 ± 2.3b	159.3 ± 5.6a	194.3 ± 7.6b	110.3 ± 5.6b	39.2 ± 1.0a	38.8 ± 1.3b	25.9 ± 1.3b
AWDN2	121.0 ± 5.9a	131.0 ± 5.9a	82.0 ± 1.9a	160.2 ± 6.8a	210.2 ± 9.8a	128.2 ± 4.7a	38.5 ± 1.1a	48.5 ± 2.1a	31.5 ± 1.1a
AWDN3	102.7 ± 3.5b	135.7 ± 6.1a	80.7 ± 2.1a	151.1 ± 3.7b	216.1 ± 8.6a	129.1 ± 3.9a	32.3 ± 0.9b	47.3 ± 1.9a	32.3 ± 1.6a

CI and AWD represent conventional flooding irrigation and alternate wetting and moderate drying irrigation, respectively. N1, N2, and N3 represent 100% CU, 60% CRNF + 40% CU, and 100% CRNF at an equivalent N rate of 240 kg ha^−1^, respectively. Values are mean of two years and three replicates, and values (means ± standard error, n =6) followed by different letters within a column are significantly different at the probability level of 0.05.

### 3.1 LAI and *SPAD* values

Under all treatments, the LAI reached the maximum in the heading stage and then declined until the maturity stage. The LAI in the tillering stage was significantly higher (increased by 12.4%–17.8%) in N1 and N2 plots compared with N3 plots under AWD and CI (**Figure 2**). The LAI in the jointing stage was significantly higher in AWD-treated plots irrespective of the N treatments. Additionally, the N1 and N2 had a higher LAI in the jointing stage than the N3 under the two irrigation regimes. The LAI in the heading, filling, and maturity stages was 21.4%–45.6% higher in AWD than CI, irrespective of N treatments. Furthermore, the N2 and N3 resulted in a higher LAI in the three aforementioned growth stages than the N1 under the two irrigation regimes. AWDN2 treatment led to the highest LAI in the five measured growth stages (**Figure 2**). *SPAD* values in the measured growth stages under different treatments showed similar variations compared with the LAI in the corresponding stages (**Figure 3**).

### 3.2 Activities of SOD, POD, and CAT

The activities of SOD, POD, and CAT under all treatments were the highest in the heading stage (**Table 2**). The N1 and N2 groups had the highest activities of SOD, POD, and CAT (increased by 9.2%–21.3%) in the jointing stage compared with the N3 group under CI and AWD. Additionally, AWD plots had higher activities of SOD, POD, and CAT (increased by 11.9%–35.7%) compared with CI plots, irrespective of the N treatments. Also, AWD had higher activities of SOD, POD, and CAT in the latter growth (heading and maturity) stages. Additionally, N2 and N3 resulted in higher activities of SOD, POD, and CAT (increased by 9.4%–22.7%) than N1 under the two irrigation regimes. The highest activities of SOD, POD, and CAT were found under AWDN3 and AWDN2 treatments, whereas the lowest activities of SOD, POD, and CAT were observed under CIN1 treatment (**Table 2**).

### 3.3 Activities of NR, GS, and GOGAT

The activities of NR, GS, and GOGAT showed a rise-and-fall trend in the growing season, peaking in the heading stage before decreasing (**Table 3**). AWD had higher activities of NR, GS, and GOGAT in the jointing stage (increased by 12.4%–31.7%) compared with CI at each N rate. Additionally, N1 and N2 had higher activities of NR, GS, and GOGAT (increased by 13.5%–22.8%) than N3 under CI and AWD. The CIN3 treatment exhibited the lowest activities of NR, GS, and GOGAT. AWD plots had higher activities of NR, GS, and GOGAT (increased by 17.8%–40.1%) in the heading and maturity stages compared with CI plots. N2 and N3 resulted in higher activities of NR, GS, and GOGAT (increased by 14.5%–36.3%) than N1 under AWD and CI. The AWDN2 and AWDN3 treatment groups had the highest activities of NR, GS, and GOGAT, whereas these activities were the lowest in the CIN1 treatment group (**Table 3**).

### 3.4 *P*_n_

The *P*_n_ values under all treatments were the highest in the heading stage (**Figure 4**). The *P*_n_ values were significantly (12.8%–28.6%) greater in N1 and N2 plots compared with N3 plots in the jointing stage, irrespective of the irrigation treatment. Additionally, AWD plots had higher *P*_n_ (increased by 15.0%–30.1%) than CI plots at each N rate. The *P*_n_ was significantly higher (increased by 13.4%–22.5%) in N2 and N3 plots compared with N1 plots in the heading and maturity stages, irrespective of the irrigation regimes; N2 had higher *P*_n_ than N3 under AWD. Moreover, AWD increased *P*_n_ compared with CI. Hence, AWDN2 treatment provided the highest *P*_n_ (**Figure 4**).

### 3.5 MDA and soluble protein contents

The MDA content peaked in the maturity stage (**Figure 5**). It was significantly higher (16.7%–48.2%) in N3 plots compared with N1 and N2 plots in the jointing stage, irrespective of the irrigation treatment. Additionally, CI plots had higher MDA content (increased by 16.8%–32.1%) than AI plots under each N treatment. The MDA content was significantly higher (10.1%–21.9%) in N1 plots compared with N2 and N3 plots in the heading and maturity stages, irrespective of the irrigation regimes. Moreover, CI increased the MDA content compared with AWD. Hence, AWDN2 and AWDN3 treatments led to the lowest MDA content, whereas CIN1 treatment led to the highest MDA content (**Figure 5**).

The soluble protein content under all treatments was the highest in the heading stage (**Figure 6**). The soluble protein content was significantly higher (13.7%–28.6%) in N1 and N2 plots compared with N3 plots in the jointing stage under the two irrigation regimes. Additionally, AWD plots had higher soluble protein content (increased by 11.1%–32.0%) than CI plots at each N rate. The soluble protein content was significantly higher in N2 and N3 plots (increased by 9.8%–25.4%) compared with N1 plots in the heading and maturity stages, irrespective of the irrigation treatment. Moreover, AWD increased the soluble protein content by 13.1%–32.8% compared with CI. Hence, AWDN2 and AWDN3 treatments yielded the highest soluble protein content, whereas CIN1 treatments yielded the lowest soluble protein content (**Figure 6**).

### 3.6 Economic benefits

The fertilizer cost was the highest in the N3 plot, followed by the N2 plot, and the lowest in N1 in the 2 years (**Table 4**). AWD significantly lowered labor costs (reduced by 17.3%–24.5%) compared with CI under each N treatment. Additionally, N1 increased the frequency of N fertilization (up to three times), and thus labor cost (increased by 6.7%–8.1%), compared with N2 and N3 under AWD and CI. For the CI plots, the rice field experienced irrigation events seven times in the 2 years, and the total amount of irrigation was 430 mm in 2020 and 414 mm in 2021. For the AWD plots, the total amount of irrigation was 275 mm in 2020 (irrigation four times) and 249 mm in 2021 (irrigation three times). As a result, AWD lowered water costs (reduced by 36.0%–39.8%) compared with CI. AWD significantly increased net income compared with CI under each N treatment. N2 and N3 had a higher net income than N1 under AWD and CI; N2 increased the net income compared with N3 under AWD. Consequently, AWDN2 treatment achieved the highest net income (13,907.1 CNY ha^−1^ in 2020 and 14,085.7 CNY ha^−1^ in 2021), whereas CIN1 treatment resulted in the lowest total income (**Table 4**).

**Table 4:** Economic efficiency of rice under different treatments in 2020 and 2021 (CNY ha^−1^).

Treatment	Input				Output (total income)	Net income
	Fertilizer cost	Labor cost	Water fee	Other costs		
2020						
CIN1	1,304.3	2,080.0	430.0	4,870.0	14,833.0d	6,148.7e
CIN2	2,165.5	1,960.0	430.0	4,870.0	18,943.6c	9,518.1d
CIN3	2,679.0	1,960.0	430.0	4,870.0	18,891.6c	8,952.6d
AWDN1	1,304.3	1,720.0	275.0	4,870.0	18,694.0c	10,524.7c
AWDN2	2,165.5	1,600.0	275.0	4,870.0	22,817.6a	13,907.1a
AWDN3	2,679.0	1,600.0	275.0	4,870.0	20,358.0b	10,934.0b
2021						
CIN1	1,304.3	2,080.0	414.0	5,230.0	15,314.0c	6,285.7e
CIN2	2,165.5	1,960.0	414.0	5,230.0	19,448.0b	9,678.5d
CIN3	2,679.0	1,960.0	414.0	5,230.0	19,679.4b	9,396.4d
AWDN1	1,304.3	1,600.0	249.0	5,230.0	19,188.0b	10,804.7c
AWDN2	2,165.5	1,480.0	249.0	5,230.0	23,210.2a	14,085.7a
AWDN3	2,679.0	1,480.0	249.0	5,230.0	22,191.0a	12,553.0b

Per kg of CU (46% N) and CRNF (43% N) was 2.5 and 4.8 yuan, respectively. Water cost was 1.0 CNY m^−3^. Rice price was 2.5 CNY kg^−1^. Unit labor cost was 120.0 CNY d^−1^. CI and AWD represent conventional flooding irrigation and alternate wetting and moderate drying irrigation, respectively. N1, N2, and N3 represent 100% CU, 60% CRNF + 40% CU, and 100% CRNF at an equivalent N rate of 240 kg ha^−1^, respectively. Data are presented as mean values (n = 3); values followed by different letters within a column and same year are significantly different at the probability level of 0.05.

### 3.7 Grain yield

**Figure 7:**
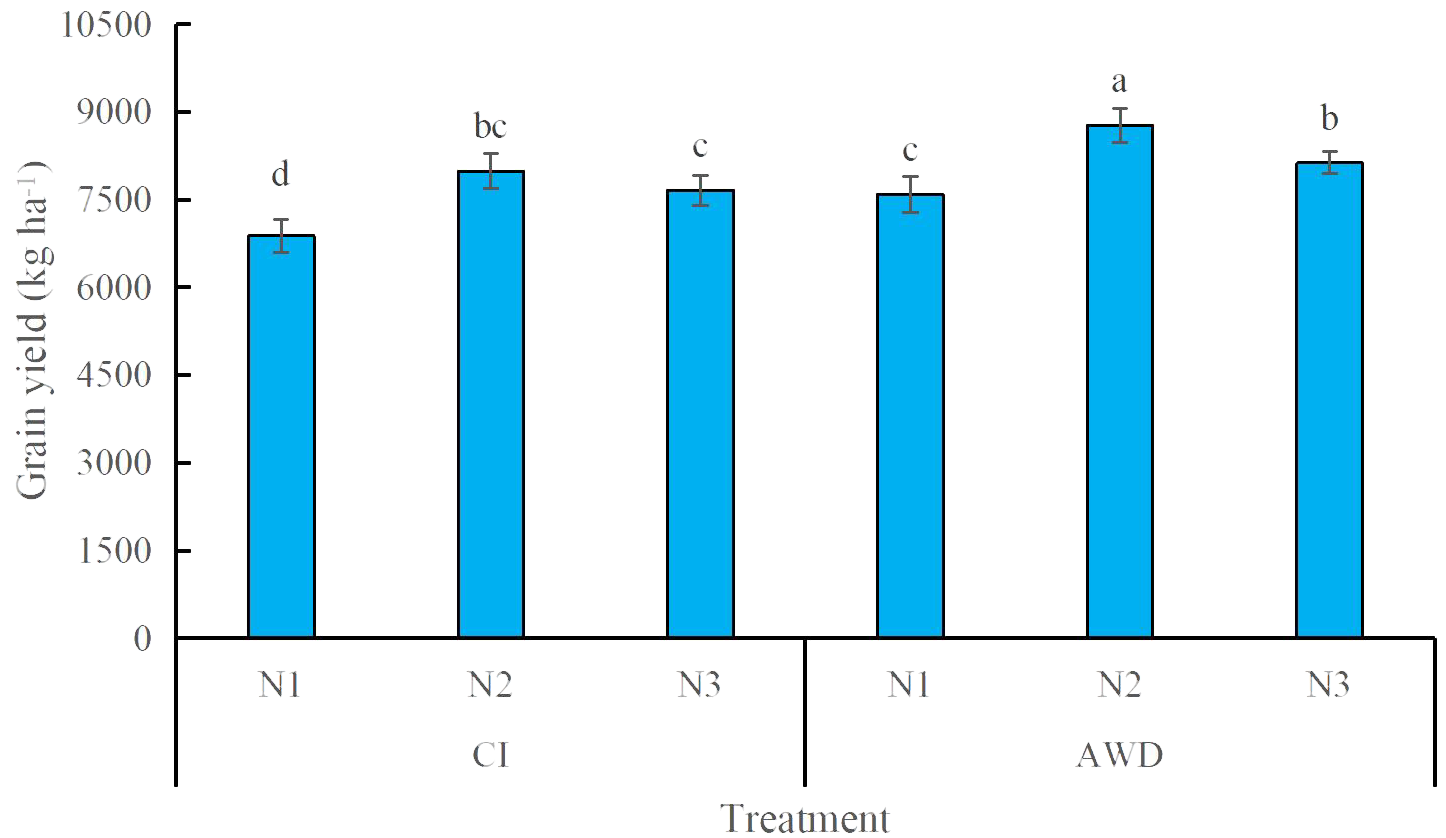
Grain yield of rice leaves affected by different water and nitrogen management strategies. CI and AWD represent conventional flooding irrigation and alternate wetting and moderate drying irrigation, respectively. N1, N2, and N3 represent 100% CU, 60% CRNF + 40% CU, and 100% CRNF at an equivalent N rate of 240 kg ha^−1^, respectively. Values (mean ± standard error, n = 6) are mean of 2 years and three replicates. Means by different letters are significantly different at *p* < 0.05.

AWD significantly increased grain yield compared with CI, irrespective of N treatments (**Figure 7**). N1 resulted in the lowest grain yield under the two irrigation regimes. Also, N2 rather than N3 provided a higher grain yield under AWD. However, the CIN1 treatment resulted in a low yield (**Figure 7**).

## 4 Discussion

The effect of AWD, CRNF, and their interaction on rice yield and resource use efficiency has been evaluated previously (Ye et al., 2013; Li et al., 2018; Zhang et al., 2021; Yan et al., 2022; Fu et al., 2024). Moreover, it has been shown that AWD combined with the mixed application of CRNF and CU can improve grain yield, WUE, and NUE of rice (Zhang et al., 2021). Nevertheless, the physiological mechanism underlying this effect remains largely unknown. Also, the combined impact of AWD and the blended application of CRNF and CU on economic benefits in rice is unclear. This study found that the irrigation regimes and the N fertilizer compounding modes worked together to affect *P*_n_, LAI, and *SPAD* value; activities of SOD, POD, CAT, NR, GS, and GOGAT; and contents of MDA and soluble protein in rice leaves. Moreover, they influenced the total and net income in rice. Obviously, AWD interacted with N2 or N3 to provide a positive synergistic interaction for delaying leaf senescence compared with N1 under the same watering regime. Moreover, the results indicated that AWD combined with N2 could delay leaf senescence by improving photosynthesis, antioxidant defense system, osmoregulation, and N assimilation, contributing to high grain yield and net income in rice.

### 4.1 Effects of AWD and N management strategies on the leaf senescence characteristics of rice

Leaf senescence is characterized by a reduction in the LAI and *SPAD* values. Chlorophyll is a light-harvesting molecule, and its content (*SPAD* value) indicates the potential of the plants to yield photosynthate by absorbing light energy (Ren et al., 2023). The LAI, which signifies the photosynthetic capacity of leaves, directly influences dry matter accumulation and yield formation (Gitelson et al., 2014). In the present study, AWD resulted in a higher LAI (**Figure 2**) and *SPAD* value in the heading, filling, and maturity stages (**Figure 3**), suggesting that alternate wetting and drying irrigation can sustain normal functioning of leaves in rice in the post-growth stages. Three possible scenarios may account for this phenomenon. First, AWD enhanced gas exchange between soil and atmosphere due to wetting and drying cycles, creating an oxygen-rich area around the rhizosphere. This expedited the mineralization of soil organic matter and restrained the immobilization of soil N (Cao et al., 2022). Second, AWD is known to improve the morphological and physiological characteristics of roots in rice (Xu et al., 2018; Zhang et al., 2021), enhancing the vitality of roots to absorb soil water and nutrients (Wang et al., 2016). This leads to high relative water content in leaves (Yang et al., 2022) and active N metabolism (Yan et al., 2022), corresponding to the enhanced *P*_n_ (**Figure 4**) and activities of NR, GS, and GOGAT (**Table 4**). Third, AWD decreased MDA content (**Figure 5**) and increased the activities of SOD, POD, and CAT (**Table 4**), suggesting better ROS scavenging in AWD-treated plants. Also, N2 and N3 compared with N1 resulted in a greater LAI (**Figure 2**) and *SPAD* value in the post-growth stages (**Figure 3**). This could be related to the discharge characteristics of CRNF as “peak value cutting and valley value filling”. In other words, a continuous and sustained N release occurred throughout the rice-growing season involving CRNF plots (Peng et al., 2014). The enhanced soil N availability promotes the development of a strong and vigorous root system (Ye et al., 2013; Qi et al., 2023b), enhancing water and N utilization (Zhang et al., 2021). CU is rapidly hydrolyzed by urease when applied to the soil (Guo et al., 2021). The excessive decomposition of CU leads to its incomplete utilization by crops. Moreover, CU fertilization (basal application + tillering topdressing) for N1 in this study usually overlapped with the plum rain season (well known for continuous overcast and rains in the middle and lower Yangtze River) (Qi et al., 2020). The heavy rainfall induced enormous N losses even at a moderate N rate (Li et al., 2018; Qi et al., 2023a). This could lead to soil N deficiency in the latter growth stages for N1, lowering LAI and *SPAD* values. Hence, the mixed application of CRNF and CU (as in N2) increased soil N content in various growth stages (Zhang et al., 2021; Qi et al., 2023a). This was supported by the higher *P*_n_, LAI, and *SPAD* values and activities of NR, GS, and GOGAT observed in the jointing stage in the N2 plots. In addition, CRNF blended with CU obviously improved soil NO_3_^−^–N and NH_4_^+^–N contents in the plow layer (0–40 cm) across the rice-growing season (Zheng et al., 2016). This was in line with the results of other studies on the maize leaf senescence characteristics under the mixed application of CRNF and CU (Guo et al., 2021; Yu et al., 2023). AWDN2 and AWDN3 treatments provided the highest LAI and *SPAD* values in the post-growth stages.

Plant senescence is closely related to the ability to scavenge the ROS (Choudhury et al., 2017). The activities of SOD, POD, and CAT can reflect a plant’s ability to scavenge ROS. CAT and POD metabolize H_2_O_2_, whereas SOD catalyzes the disproportionation of O2^−^ to H_2_O_2_ (Ahmed et al., 2002). MDA can combine with proteins of the cell membrane structure, inactivating the related proteins; its content can be used to reflect the degree of lipid peroxidation (Biemelt et al., 2000). Compared with CI, AWD had higher SOD, POD, and CAT activities, whereas the MDA content was smaller, suggesting enhanced ROS scavenging ability in alternate wetting and drying irrigation. This was consistent with the previously published findings (Fu et al., 2023, Fu et al., 2024). Notably, the root systems in AWD grow better in oxygen-enriched rhizosphere (Xu et al., 2018), leading to various positive physiological effects based on ABA signaling (Liu et al., 2005). Moreover, appropriate N fertilization is useful for SOD, POD, and CAT activities (Zhang and Li, 2007). Compared with N1, N2 and N3 provided higher SOD, POD, and CAT activities and lower MDA content, suggesting better scavenging in CRNF-treated plants. This was also consistent with the findings of Yu et al. (2023) on maize. CRNF leads to better soil N availability (Zheng et al., 2016), improving root growth and vitality to enhance water and nutrient utilization (Yan et al., 2022). Additionally, optimal N management strategies upregulate antioxidant enzyme activity by enhancing the expression of related genes (Ozcubukcu et al., 2014). Therefore, AWD combined with N2 and N3 resulted in the highest SOD, POD, and CAT activities and lowest MDA contents in rice leaves.

Soluble proteins are involved in various metabolic activities, and hence, a decrease in their levels reflects the degree of leaf senescence. The levels of *P*_n_ can be used to reflect the photosynthetic capacity (Li et al., 2020). GS, GOGAT, and NR are the key metabolism-related enzymes involved in the N assimilation of rice plants (Sun et al., 2015; Wang et al., 2016). AWD increased the activities of GS, GOGAT, and NR (**Table 4**) and soluble protein content (**Figure 6**) compared with CI, resulting in a higher *P*_n_ (**Figure 4**) in various growth stages. This suggested that alternate wetting and drying irrigation improved the soil aeration conditions and enhanced soil N availability to the root system, producing more assimilates and enhancing the assimilation of inorganic N and photosynthetic capability, thus establishing a solid foundation for higher grain yield (**Figure 7**). Enhanced *P*_n_ was directly associated with higher soluble protein content, LAI, and *SPAD* values under AWD (Wang et al., 2016). Moreover, a higher LAI was always accompanied by a higher relative water content of leaves (Li et al., 2010). The rise in water status inhibited ABA production in leaves, increasing stomatal conductance (Bahrun et al., 2002). Stomata opening can increase the *P*_n_ levels (Kang and Zhang, 2004). Alternately, the root oxidation capacity, absorption area, and cytokinin content were significantly positively correlated with N metabolism-related enzyme activity in rice leaves (Fu et al., 2024). AWD increased rhizosphere oxygen content, resulting in high root oxidation capacity and root cytokinin content (Qi et al., 2023b), thereby upregulating photosynthetic acclimation (Gu et al., 2017). This was supported by higher N accumulation in various growth stages in AWD-treated plants (**Supplementary Table S1**). The enhanced N uptake in the post-growth stage was accompanied by lasting greenness in plant leaves, providing more metabolites to the roots, thus contributing to vigorous root growth (Qi et al., 2023b) and high water–N absorptive capacity (Xu et al., 2018). In turn, the shoot growth and grain yield were enhanced due to AWD (Wang et al., 2016). Compared with N1, N2 and N3 significantly increased the activities of GS, GOGAT, and NR (**Table 4**), soluble protein content (**Figure 6**), and *P*_n_ (**Figure 4**) in the heading and maturity stages, suggesting that CRNF treatments can enhance the assimilation of inorganic N and photosynthetic capability in rice. This established a solid foundation for enhancing grain filling (Sun et al., 2012), leading to high grain yield (**Figure 7**). The enhanced contents of nitrate N and ammonium N in soil under CRNF treatments accounted for the improvement in plant N nutrition (Guo et al., 2021). Alternatively, a close relationship existed between soil N availability and the activities of key N assimilatory enzymes of rice leaves in the post-growth stage (Yang et al., 2012). In addition, the CRNF promoted the transfer of N from the organs to the grains, increasing grain N content (Wang et al., 2016). Therefore, AWD combined with N2 and N3 improved N metabolism and photosynthetic capacity, achieving a higher N accumulation and grain yield.

### 4.2 Effects of AWD and N management strategies on the economic benefits of rice

The input cost and yield benefits are two important factors determining the economic efficiency of rice. Grain yield is significantly and positively correlated with total income. The application of N fertilizer and irrigation regime are conjunct to influence crop yield and thus the economic performance (Yuan et al., 2022). In this study, N1 resulted in the lowest grain yield and, thus, low total income. Although N2 and N3 led to higher total income, the higher total income from N3 was partially offset by higher fertilizer costs. The highest net income in the 2 years was obtained under AWDN2 treatment (13,907.1 CNY ha^−1^ in 2020 and 14,085.7 CNY ha^−1^ in 2021). The reduced water cost (irrigation amount) and labor cost (irrigation frequencies) accounted for the enhanced net income. Furthermore, AWDN2 resulted in smaller N loss via runoff and leaching (Qi et al., 2023a). Thus, alternate wetting and drying irrigation combined with the mixed application of CRNF and CU is recommended for delaying leaf senescence while realizing considerable ecological and economic benefits. However, the impacts of different ratios of CRNF and CU on leaf senescence remain unclear. Also, the findings of this study should be validated in other regions with different weather conditions. The mechanisms underlying delayed leaf senescence under water and N coupling should be clarified from the perspective of rhizosphere microorganisms in the future.

## 5 Conclusions

AWD interaction with CRNF or mixed application of CRNF and CU delayed leaf senescence by improving photosynthesis, antioxidant defense system, osmoregulation, and N assimilation, thus achieving higher grain yield. Moreover, AWD reduced water costs by decreasing both the amount and frequency of irrigation. Also, the mixed application of CRNF and CU lowered fertilization costs compared with using 100% CRNF alone. Thus, AWD combined with the mixed application of CRNF and CU not only delayed leaf senescence but also improved the net income of rice. Thus, the present study provided the theoretical basis to sustain the beneficial effects of the blended use of CRNF and CU on rice growth and resource utilization under AWD.

## Data Availability

The original contributions presented in the study are included in the article/**Supplementary Material**. Further inquiries can be directed to the corresponding author.

## Supplementary material

Supplementary Table 1 Nitrogen (N) uptake by rice (kg ha^-1^) as affected by different water and nitrogen management strategies. CI and AWD represent conventional flooding irrigation and alternate wetting and moderate drying irrigation, respectively. N1, N2, and N3 represent 100% CU, 60%CRNF + 40% CU, and 100% CRNF at an equivalent N rate of 240 kg ha^−1^, respectively. Values are mean of 2 years and three replicates. Means within a column followed by different letters are significantly different at *P<* 0.05.
