# Supplementary material for: Leaf senescence characteristics and economic benefits of rice under alternate wetting and drying irrigation and blended use of polymer-coated and common urea
Source: Front Plant Sci. 2024 Dec 24;15:1444819. doi: 10.3389/fpls.2024.1444819 (PMC11703807; doi:10.3389/fpls.2024.1444819)
Supplement: Supplementary Table 1 — Nitrogen (N) uptake by rice (kg ha-1) as affected by different water and nitrogen management strategies. CI and AWD represent conventional flooding irrigation and alternate wetting and moderate drying irrigation, respectively. N1, N2, and N3 represent 100% CU, 60%CRNF + 40% CU, and 100% CRNF at an equivalent N rate of 240 kg ha−1, respectively. Values are mean of 2 years and three replicates. Means within a column followed by different letters are significantly different at P< 0.05. [file Table1.docx]

**Table S1** Nitrogen (N) uptake by rice (kg ha^-1^) as affected by different water and nitrogen management strategies

| Irrigation method | Fertilizer management | Jointing | Heading | Filling | Maturity |
| --- | --- | --- | --- | --- | --- |
| CI | N1 | 60.19 d | 87.62 c | 124.54 c | 144.66 d |
|  | N2 | 75.14 b | 97.85 b | 158.55 ab | 171.81 b |
|  | N3 | 68.59 c | 98.17 b | 148.82 b | 170.79 b |
| AWD | N1 | 78.13 ab | 99.81 b | 143.51 b | 158.32 c |
|  | N2 | 82.91 a | 110.23 a | 162.15 a | 181.02 a |
|  | N3 | 71.06 c | 104.35 ab | 156.00 ab | 172.72 b |

Note: CI and AWD represent conventional flooding irrigation and alternate wetting and moderate drying irrigation, respectively. N1, N2, and N3 represent 100 % CU, 60 %CRNF + 40% CU, and 100 % CRNF at an equivalent N rate of 240 kg ha^−1^, respectively. Values are mean of 2 years and three replicates. Means within a column followed by different letters are significantly different at *P* < 0.05.
